# Effects of Transcranial Direct Current Stimulation, Transcranial Pulsed Current Stimulation, and Their Combination on Brain Oscillations in Patients with Chronic Visceral Pain: A Pilot Crossover Randomized Controlled Study

**DOI:** 10.3389/fneur.2017.00576

**Published:** 2017-11-01

**Authors:** Aurore Thibaut, Cristina Russo, Aura Maria Hurtado-Puerto, Jorge Leon Morales-Quezada, Alícia Deitos, John Christopher Petrozza, Steven Freedman, Felipe Fregni

**Affiliations:** ^1^Neuromodulation Center, Spaulding Rehabilitation Hospital, Department of Physical Medicine and Rehabilitation, Harvard Medical School, Boston, MA, United States; ^2^Coma Science Group, GIGA-Research, University and University Hospital of Liege, Liege, Belgium; ^3^Department of Psychology, Milan Center for Neuroscience-NeuroMi, University of Milano-Bicocca, Milano, Italy; ^4^Laboratory for Neuropsychiatry and Neuromodulation, Transcranial Magnetic Stimulation Clinical Service, Department of Psychiatry, Massachusetts General Hospital, Boston, MA, United States; ^5^Center for Integrative Medicine Research, Beth Israel Deaconess Medical Center, Harvard Medical School, Boston, MA, United States; ^6^Post-Graduate Program in Medical Sciences, School of Medicine, Universidade Federal do Rio Grande do Sul (UFRGS), Porto Alegre, Brazil; ^7^Laboratory of Pain and Neuromodulation, Hospital de Clínicas de Porto Alegre (HCPA), Porto Alegre, Brazil; ^8^Department of Gynecology, Massachusetts General Hospital, Harvard Medical School, Boston, MA, United States; ^9^Division of Translational Research, Beth Israel Deaconess Medical Center, Boston, MA, United States

**Keywords:** chronic visceral pain, pain, transcranial direct current stimulation, transcranial pulsed current stimulation, non-invasive brain stimulation, electroencephalogram

## Abstract

**Objective:**

Chronic visceral pain (CVP) syndromes are persistently painful disorders with a remarkable lack of effective treatment options. This study aimed at evaluating the effects of different neuromodulation techniques in patients with CVP on cortical activity, through electreocephalography (EEG) and on pain perception, through clinical tests.

**Design:**

A pilot crossover randomized controlled study.

**Settings:**

Out-patient.

**Subjects:**

Adults with CVP (>3 months).

**Methods:**

Participants received four interventions in a randomized order: (1) transcranial pulsed current stimulation (tPCS) and active transcranial direct current stimulation (tDCS) combined, (2) tPCS alone, (3) tDCS alone, and (4) sham condition. Resting state quantitative electroencephalography (qEEG) and pain assessments were performed before and after each intervention. Results were compared with a cohort of 47 healthy controls.

**Results:**

We enrolled six patients with CVP for a total of 21 visits completed. Compared with healthy participants, patients with CVP showed altered cortical activity characterized by increased power in theta, alpha and beta bands, and a significant reduction in the alpha/beta ratio. Regarding tES, the combination of tDCS with tPCS had no effect on power in any of the bandwidths, nor brain regions. Comparing tPCS with tDCS alone, we found that tPCS induced higher increase in power within the theta and alpha bandwidths.

**Conclusion:**

This study confirms that patients with CVP present abnormal EEG-indexed cortical activity compared with healthy controls. Moreover, we showed that combining two types of neurostimulation techniques had no effect, whereas the two interventions, when applied individually, have different neural signatures.

## Introduction

Visceral pain results from nociceptor activation in thoracic, pelvic, or abdominal visceral organs (1). While acute pain has the vital role of preventing tissue damage, maladaptive processes may convert it into chronic (2), leading to chronic visceral pain (CVP), a condition featured by several maladaptive neural changes, one of them being central sensitization (3). The neural mechanisms of central sensitization in chronic neuropathic conditions are partially understood (4, 5). For instance, Simis et al. showed that patients with pelvic pain had significantly lower levels of N-acetylaspartate (NAA) and creatinophosphocreatine (Cr), reflecting loss of neuronal integrity in the primary motor cortex compared with healthy participants. This evidence also highlights the process of maladaptive plasticity in neural circuits involved in pain modulation (6). Notwithstanding, there is limited evidence showing neural correlates of pain-induced central sensitization in CVP. In addition, the investigation of cortical oscillation patterns by means of electroencephalography (EEG) is still at its infancy in patients with CVP.

This paucity of knowledge regarding neural correlates of CVP further undermines treatment interventions. CVP is characterized by high level of disability and discomfort (7–9) which affect patients’ quality of life and represent an important clinical problem and socioeconomic burden for societies (9–13). The lack of effective treatments, in turn, favors the excessive use of opioids (and their side effects), potentially leading to abuse (14, 15). Therefore, it is essential to develop new therapeutic options for CVP. Preliminary studies have shown that transcranial electrical stimulation (tES), in particular transcranial direct current stimulation (tDCS), may help managing pain. tDCS, in fact, by influencing neuronal cortical activity (16), has been proven capable of inducing clinical improvements in several chronic conditions (17, 18), such as fibromyalgia (19), abdominal (20), and refractory pelvic (21) pain. Recently, another tES technique, namely, transcranial pulsed current stimulation (tPCS) has gained increasing attention in experimental settings (22–24). tPCS, using randomly pulsed alternating current within a determined frequency range, is thought to reach cortical and subcortical brain regions such as the midbrain, pons, thalamus, insula, and the hypothalamus (25). Therefore, also tPCS, thanks to its mechanisms of action, could represent a promising tool for the treatment of pain, as it is potentially able to influence deeper structures involved in chronic pain.

In this scenario, we conducted a pilot study with a twofold aim: first, we wanted to assess baseline cortical activity of CVP patients; results are compared with those obtained by a normative sample of neurological healthy participants, published elsewhere (26). Second, we sought to assess the effects of two tES interventions, on cortical activity by means of EEG. We also provided preliminary data regarding the effects of such intervention on clinical pain assessments.

## Materials and Methods

### Participants

The inclusion criteria were as follows: (1) age between 18 and 65; (2) history of visceral pain for at least 3 months; (3) an average pain ≥4 in the past 3 months, as measured by the visual analog scale (VAS); (4) no history of neurologic or psychiatric conditions and no current unstable medical conditions; (5) no contraindications to tES; and (6) no current pregnancy. The study was approved by the Institutional Review Board of Spaulding Rehabilitation Hospital and performed in accordance with the Declaration of Helsinki. Healthy subjects’ cohort data have been published elsewhere (26).

### Study Design

We conducted a pilot randomized, double-blind, sham-controlled crossover trial. Participants received the following four interventions in a randomized order separated by a minimum of 5 days: (1) active tPCS/active tDCS; (2) active tPCS/sham tDCS; (3) sham tPCS/active tDCS, and (4) sham tPCS/sham tDCS. Each stimulation condition was preceded and followed by EEG and clinical assessments.

### Transcranial Direct Current Stimulation

Transcranial direct current stimulation was delivered with the anode electrode positioned over the left primary motor cortex (M1) and the cathode electrode over the contralateral right supra orbital region (Soterix Medical, NY, USA). Stimulation parameters were as follows: 20 min at 2 mA, 30-s fade-in and fade-out.

### Transcranial Pulsed Current Stimulation

We used an investigational, custom-made, battery-powered and high-frequency tPCS device (Lab 8Tron AG) that delivered a quadratic biphasic alternating current using periauricular ear-clip electrodes. Stimulation parameters for tPCS were as follows: 20 min of stimulation at a fixed current intensity of 2 mA and a random noise frequency of 6–10 Hz [as previously described (23)]. For sham conditions (both tDCS and tPCS), stimulation parameters were the same, but the device turned off automatically after 30 s (27).

### Quantitative Electroencephalography (qEEG) Recording, Analysis of Power and Interhemispheric Coherence

We used a 64-channel, high-density Electrical Geodesic Incorporated EEG device (Electrical Geodesics, OR, USA). EEG was recorded for 6 min eyes-closed. Data were sampled at 250 Hz, amplified and filtered using a bandpass of 0.1–70 Hz. For offline analysis, we used a low-pass filter of 40 Hz and high-pass of 1 Hz, followed by manual artifact detection and rejection by a blind assessor. Power and coherence were calculated using EEGLab (28) and MATLAB (MATLAB R2012a; The MathWorks Inc., Natick, MA, USA). Fast Fourier transformation (averaged windows of 5 s with 50% overlap) was used to calculate power (μV2) for the EEG bands theta (4–8 Hz), alpha (8–13 Hz) and beta (13–30 Hz), and the sub-bands low-alpha (8–10 Hz) and high-alpha (10–13 Hz). We also determined the alpha/beta power ratio. The signal from adjacent electrodes was averaged to represent frontal, central, parietal, temporal, and occipital areas.

We calculated interhemispheric coherence for these bands and sub-bands using two different electrode pairs: E19–E56 and E14–E57, located in the frontotemporal junction and including their reciprocal location in the contralateral hemisphere. Welch’s averaged modified periodogram method was used to find the estimated coherence of signal *x* and *y*, representing each electrode site.

### Clinical Assessments

Visual analog scale for pain, anxiety, depression, stress, and sleepiness was collected. Von Frey Hair Assessment (North Coast Medical, Inc., Morgan Hill, CA, USA), comprised of monofilaments (0.008–300 g) was used to determine subjects’ perception threshold. This assessment was performed on the patient’s most painful region and over the ipsilateral hand to serve as a control. Pressure pain threshold (PPT) was also performed to measure subjects’ pain threshold (Commander Algometer, JTECH Medical, UT, USA). PPT was measured over the thenar eminence of both hands, namely, both ipsi- and contralateral to the most painful body area. For the evaluation of conditioned pain modulation (CPM), the same test applied for the PPT (*test-stimulus*) was repeated while the subject’s contralateral hand was immersed in cold water for 30 s (*conditioned-stimulus*).

### Statistical Analyses

To compare neurophysiological and behavioral data of healthy controls (HCs) with that of CVP patients, we used baseline data of the first visit for each participant. Continuous variables (i.e., age and years of education) were compared using a *t*-test, while gender was analyzed using a χ^2^ test. Regarding EEG, baseline rhythms for each frequency band were compared between patients and HC (26), using a Mann–Whitney *U* test.

For patients only, Kruskal–Wallis test was used to evaluate the effects of stimulation on power and coherence variables (difference between pre- and post-stimulation values) with *tES* (tPCS/tDCS, tPCS, tDCS, and Sham) as independent fixed variable. Mann–Whitney tests were applied for *post hoc* comparisons. For each clinical assessment, a Friedman’s ANOVA was performed, with *tES* (tPCS/tDCS, tPCS, tDCS, and Sham) as the main factor.

## Results

Six patients (2 males and 4 females) were included in the present study (see Table 1 for demographic and clinical data) for a total of 21 visits completed (5 patients completed the tPCS/tDCS session, 5 completed the tPCS session, 6 completed the tDCS session, and 5 completed the sham session). One patient drop out after the first visit (tDCS session) due to scheduling issues.

**Table 1:** Demographical and clinical data of the patients.

ID	Age/gender	Diagnosis	Race	Level of education	DI (months)	Pain medication
1	51/F	CP	Caucasian	College	36	Fentanyl^a^, Oxycodone^a^
2	24/M	CP	Caucasian	College	5	Oxycodone^a^, Dilaudid^a^
3*	36/F	CP	Caucasian	College	24	Paracetamol, Ibuprophen
4	33/M	CP	Caucasian	Bachelor	108	Oxycotin^a^, Oxycodone^a^
5	31/F	CP	African-American	Bachelor	61	Lidocaine, Ibuprophen, Hydromorphone^a^
6	44/F	CP	Caucasian	College	61	Tapentadol^a^

*^a^Opioids*.

*This patient underwent only one visit.

*CP, chronic pancreatitis; DI, duration of illness; F, female; ID, patients’ identification number; M, male*.

### Baseline Characteristics of Patients Compared with HC

Chronic visceral pain patients and HC were similar for age (*t* = 0.885; *p* = 0.432), gender (χ^2^ = 0.24; *p* = 0.622), and level of education (*t* = 2.47; *p* = 0.122). At baseline, CVP patients had significantly more power in theta (*p* < 0.001), alpha (*p* < 0.0001), low-alpha (*p* < 0.001), and beta (*p* < 0.001) bandwidths, compared with HC. On the other hand, alpha/beta ratio was significantly reduced in patients with CVP (*p* < 0.001) (Table 2).

**Table 2:** Power spectra.

Group	Theta (μV^2^)	Alpha (μV^2^)	Low alpha (μV^2^)	High alpha (μV^2^)	Beta (μV^2^)	Ratio α/β
CVP	0.10 ± 0.08*	0.19 ± 0.28*	0.29 ± 0.56*	0.13 ± 0.10	0.016 ± 0.01*	12.83 ± 5.9
HC	0.05 ± 0.08	0.15 ± 0.17	0.19 ± 0.23	0.12 ± 0.19	0.012 ± 0.01	18.6 ± 20.1

*Mean and SD of the baseline power spectrum for the different bandwidths in healthy controls (HC) and patients with chronic visceral pain (CVP)*.

*Significant difference between HC and CVP.

### EEG Power Analysis in Patients

#### tES Effect

**Figure 1:**
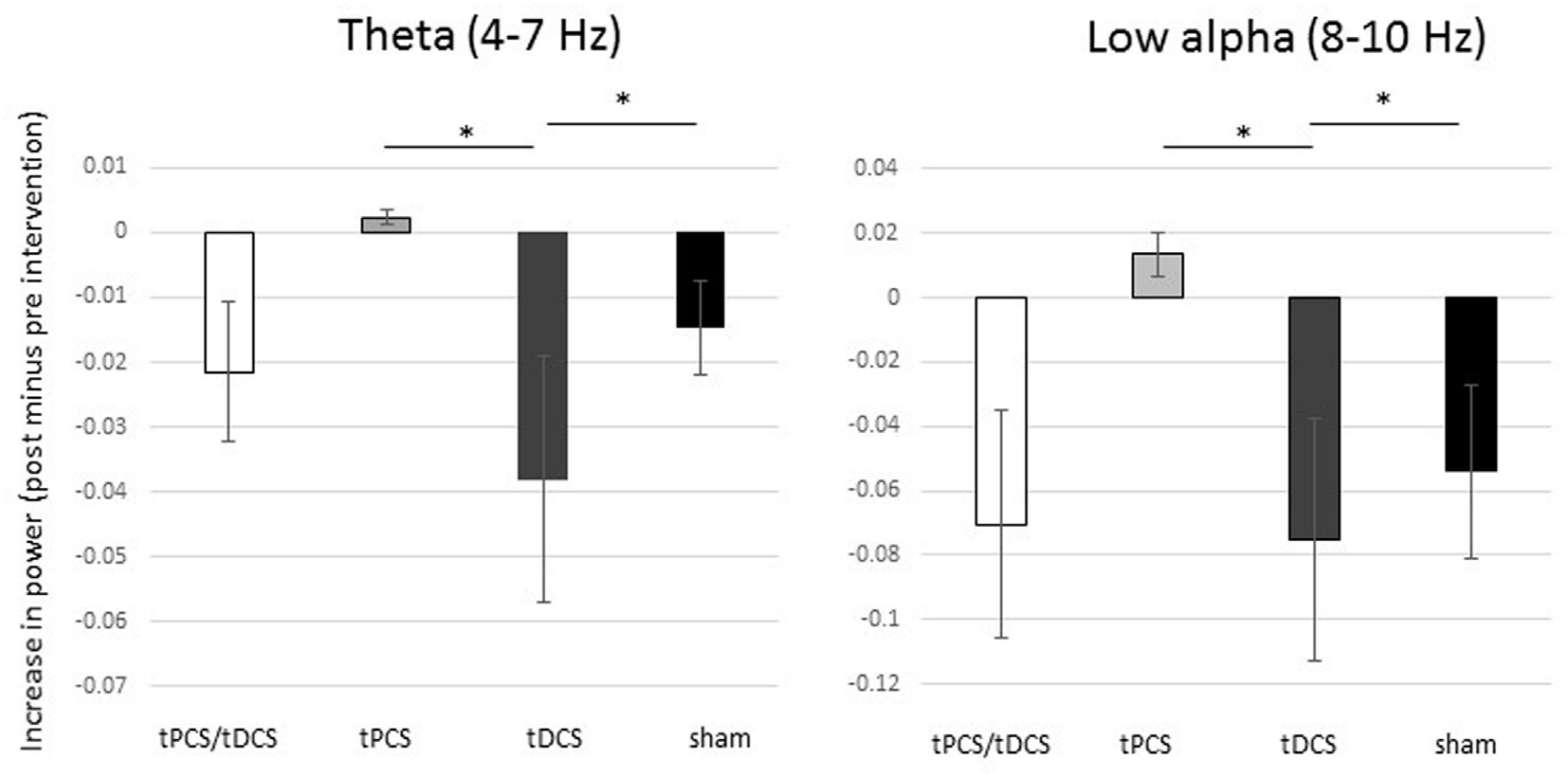
Differences in absolute power between groups. Mean differences (pre- versus post-intervention) of power for theta and low-alpha bandwidths. The bars represent the standard error. * Significant difference between groups.

**Figure 2:**
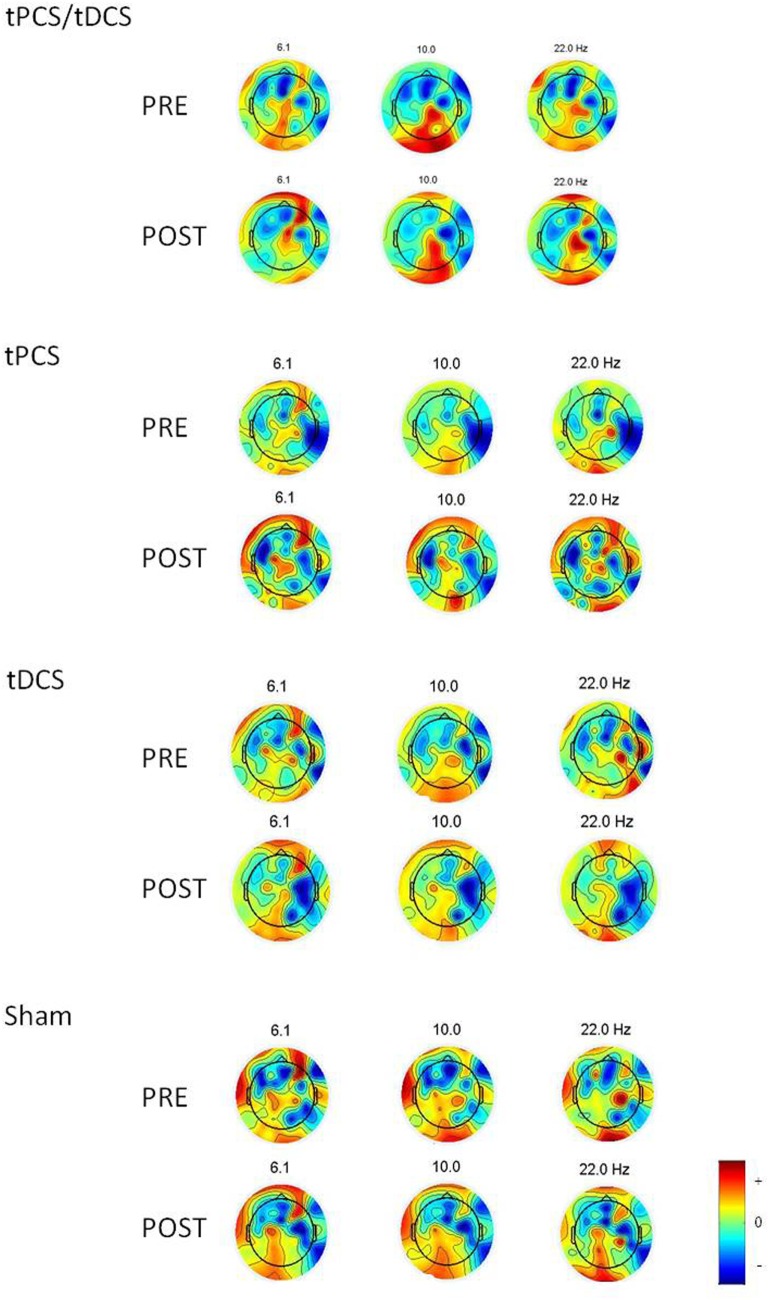
Topoplots showing the topographic distribution of the different bandwidths for a representative individual before and after each intervention. Red areas represent higher activity, while blue areas represent lower activity.

There were no differences in the baseline power spectrum in the frontal, central, parietal, temporal, or occipital brain regions (all *p*s > 0.05). After tES, there was a significant effect over all areas combined (i.e., global) for the theta bandwidth (*p* < 0.001), for the low-alpha (*p* = 0.016), high-alpha (*p* = 0.027) bandwidths, and for alpha/beta ratio (*p* < 0.001); see Figure 1. We also found a tES effect over the central and the parietal regions for the alpha/beta ratio (*p* = 0.037 and *p* = 0.028, respectively) and over the occipital region for the theta bandwidth (*p* = 0.002). No other effect was significant. Figure 2 represents the different topomaps of a representative patient (patient 1).

#### *Post Hoc* Analysis

As shown in Figure 1, combined tPCS with tDCS was not different as compared with sham for any of the frequency bands or brain regions (all *ps* > 0.05). In contrast to tDCS, tPCS induced significantly higher increase in power, in theta (*p* < 0.001) and low-alpha (*p* = 0.004) bandwidths, as well as for the alpha/beta ratio (*p* = 0.011) for global. In addition, tPCS induced a significantly higher increase in theta power over the occipital region, as compared with tDCS (*p* < 0.001).

Transcranial direct current stimulation induced higher reduction in power of theta (*p* = 0.002) and low-alpha (*p* = 0.01) bandwidths as compared with sham, as well as a reduction of alpha/beta ratio (*p* < 0.001) for global. In addition, tDCS induced a reduction of the alpha/beta ratio over the frontal region, but this decrease was significantly less important than sham (*p* = 0.023).

Transcranial pulsed current stimulation induced a decrease in power in the high-alpha bandwidth for global (*p* = 0.033) as compared with sham. Despite a significant group effect for alpha/beta ratio over the parietal region, none of the *post hoc* tests reached the significance level.

### Coherence

No group effect for any bandwidths or brain regions was found (all *p*s > 0.05).

Individual power and coherence data can be found in Supplementary Material.

### Clinical Assessments

No differences were found in either depression VAS (χ^2^ = 3.86; *p* = 0.27), anxiety VAS (χ^2^:1.5, *p* = 0.68), stress VAS (χ^2^:4.87, *p* = 0.18), sleepiness VAS (χ^2^ = 55.7, *p* = 0.12), or pain VAS (χ^2^ = 5.87, *p* = 0.12). No significant effect was found for Von Frey assessment over the painful- (χ^2^ = 6.6, *p* = 0.08), nor over the non-painful (thenar) region (χ^2^ = 2.6, *p* = 0.46) region.

Similarly, no significant differences were found for PPT (χ^2^ = 0.4; *p* = 0.94) or CPM (χ^2^ = 3.5; *p* = 0.32).

Individual clinical data can be found in Supplementary Material.

## Discussion

The present study yielded interesting findings: (1) patients with CVP display abnormal neural activity compared with healthy controls, as indexed by qEEG; (2) EEG captured cortical changes following tES, similar to what was observed in healthy controls, while no clinical improvement was noticed; (3) combining tDCS with tPCS does not induce specific changes in neural activity; (4) tDCS and tPCS have different neural signature.

So far, only few studies have investigated the neurophysiological patterns of patients with CVP. For instance, EEG measures in patients with chronic pancreatitis show an increase in power in the theta and alpha frequency bands as well (29). Present findings substantiate this altered cortical activity in patients with CVP. Our results, in fact, show an increased power in different frequencies bands (i.e., theta, low alpha, and beta) and a decrease in low versus high frequencies ratio, as compared with healthy participants (26). Therefore, this feature seems to represent a reproducible pattern in patients with chronic pain, as pointed out by a recent systematic review (30).

Baseline cortical activity could serve as a biomarker for treatment effects and as a predictor of response when using tES and other neuromodulatory techniques. As seen in patients with spinal cord injury and chronic pain, increased theta power at baseline was associated with greater response to analgesic treatment; in this case, hypnosis (31). We also noticed that the alpha/beta ratio was significantly reduced in patients with CVP, compared with healthy participants (26). Even though our sample size is small to define a neurophysiological biomarker that serves as a predictor of response to tES, EEG may represent a valuable tool for this purpose in chronic pain (29, 32, 33). For instance, it has been shown that peak alpha frequency may represent valuable biomarker for chronic pain, as this measure is not only greatly decreased in patients, compared with healthy participants, but it is also correlated with the duration of pain (29). In this context, this pilot study provides the initial data to support further studies investigating changes in brain oscillations and maladaptive plasticity in patients with CVP.

An important mechanism that deserves further investigation in the context of these results is the interaction between pain, immune system and electrical stimulation. It has been discussed before that pain may trigger also immune mechanisms in a two-way brain response to injury (34) and electrical stimulation may modulate this interaction (35). This relationship between immune system and pain may also be used to find useful biomarkers and responders. For instance, the relationship between gene expression and pain has been demonstrated in mice (36). Specifically, it has been shown that cholecystokinin is implicated in the modulation of pain sensitivity and the development of neuropathic pain in mice and, more interestingly, when the cholecystokinin receptor gene is removed, the pain sensitivity is reduced and the development of hyperalgesia is abolished (37). In this context, a combination of electrophysiology and immune system gene expression study could open new doors and new treatments options in the field of chronic pain.

While no changes were observed clinically, neurophysiologically, the combination of tDCS with tPCS did not lead to any changes in brain activity, while the application of the two techniques separately induced different cortical modifications, as already demonstrated (23, 24, 38, 39). Therefore, it is becoming increasingly apparent that the effects of tDCS and tPCS are not mediated by the same neurophysiological mechanisms (40). By reaching deeper structures, it seems feasible that tPCS could stimulate bottom–up connectivity trough thalamo-cortical circuits (40). On the other hand, tDCS seems to target a top–down cortico-thalamic pathway (41) and increases cortical excitability under the stimulating electrodes and associated cortical networks (38). It is possible that tPCS and tDCS, when applied simultaneously, would eliminate their respective effects on brain activity. This important result underscores the need of careful consideration before associating different tES techniques.

Considering each technique individually, tPCS has shown promising and reproducible results as a neuromodulatory tool given the reported effects on coherence and power (22, 23, 40). In particular, in healthy volunteers, 6–10 Hz tPCS seems to modulate brain oscillation within the spectrum contained in these frequencies (i.e., high theta and low alpha). While for tDCS, an increased power has been observed in the high beta bandwidth over temporal and parietal regions, as compared with sham.

Our results showed that tPCS induced a higher increase in power within these two specific frequency bands compared with tDCS. We also identified a decrease in power for high-alpha bandwidth after tPCS and a decrease in power for theta and low-alpha bandwidths after tDCS, as compared with sham. This reduction in EEG power may represent a cortical modulation reducing a putative pathological over-excitability in patients suffering from chronic forms of pain. In fact, baseline EEG power of the main frequency bands is increased in CVP as compared with HC, suggesting a pathological over-activity in patients, especially over the sensorimotor cortex (i.e., central region). Therefore, the decrease here found can be interpreted as a normalization of the cortical oscillations, leading to a pattern more similar to healthy conditions.

No clinical changes were observed after tDCS, tPCS, or both interventions combined. Evidence shows that repeated tES sessions, and tDCS in particular, are required to induce long-lasting and significant clinical effects in psychiatric (42), motor (43), and pain conditions (44). Particularly, a significant decrease in chronic abdominal pain was registered after 5 days of anodal tDCS over the left motor cortex and persisted 1 week later (20). Similarly, a modest reduction of pain was reported by a small group of patients after two tDCS sessions (21). Moreover, a mean pain response of 58% compared with sham was registered after 5 days of anodal stimulation of the left motor cortex in neuropathic pain (45). It is hypothesized that recurring stimulation sessions are needed to produce perceptible and lasting clinical effects due to mechanisms resembling long-term potentiation (LTP) and long-term depression (LTD). Therefore, it is quite possible that a single-stimulation session was not sufficient to induce clinical effects in this experiment. Furthermore, the EEG montage and recording, often taking up to 20 min, was performed after the end of the stimulation. This represents a gap between the end of stimulation and clinical assessments, potentially allowing for any measurable effect to fade out by the time of collection.

Present findings, although preliminary, appear promising regarding the potentiality of tES techniques in modulating pathological cortical activity in CVP. Notwithstanding, they should be considered cautiously. When studying patients suffering from chronic pain, the effect of medications on spontaneous brain oscillations should be considered. In the present study, all patients but one were under one or more opioids drugs (see Table 1). Opioid-induced slowing of the EEG has been demonstrated with different morphine derivatives such as fentanyl (46) and oxycodone (47) under controlled infusions, while tramadol an opioid-like agent favors fast EEG power spectrum responses (48), including spikes generation. Still, prolonged use of oral or topic administration of these agents might induce modulation of spontaneous brain activity measured on scalp EEG, especially, shifting the background rhythm toward the low frequencies of the spectrum. For the results presented here, medication can be considered a confounding or interacting variable which deserves better control in future studies. However, as every patient received the four conditions (except for one), we can consider that pain medication influenced brain activity identically for the four types of tES.

Regarding the sham intervention, an important placebo component was identified (i.e., increase in theta and alpha and a decrease in beta bandwidths following the sham intervention). This could be interpreted in two different ways: (1) the observed increase in theta and alpha power and decrease in high-frequency bands could be related to relaxation. A similar pattern has been observed, for instance, under meditation (49–51). Here, active tES could enhance patients’ attention and therefore does not lead to an increase in drowsiness related rhythms. Therefore, the observed modification after the sham stimulation could be due to the other active conditions that blocked the occurrence of these modifications. (2) In this population of patients, the placebo effect and expectations are important components to take into account placebo effect and is often observed in patients with chronic pain (52) and also after electrical stimulation (53). This placebo effect could also partially explain our findings following the sham stimulation. Another important limitation to consider is that this study was a pilot exploratory study, and thus the lack of significant results in some of the analyses may simply indicate lack of power for that analysis. The goal was to inform feasibility and also provide effect estimates and discuss preliminary findings to design further studies.

### Conclusion and Future Directions

With respect to tES techniques, our preliminary data suggest that the combination of tDCS with tPCS, applied simultaneously, does not lead to improvements in cortical oscillations, similarly to what has been observed in healthy population (26). We also highlighted that tDCS and tPCS influence brain activity differently, and therefore may be underlined by different neural mechanisms as previously shown (26, 40). While there are a limited amount of data on the neurophysiological reorganization in patients with CVP, our results could help designing future trials using tES as an intervention and neurophysiological assessment to evaluate its effects. EEG could represent a suitable biomarker to characterize neural states of chronic pain and to capture the immediate effects of tES (single or short-term protocol). In this scenario, EEG could further be used as a predictor of response (i.e., to differentiate treatment responders from non-responders based on their neural signature), and to better understand the neurophysiological mechanisms of tES on pain relief. The combination of clinical assessments and EEG may become a useful tool to tailor customized treatment options and improve the efficacy of tES in alleviating pain.

## Ethics Statement

The study was approved by the Institutional Review Board of Spaulding Rehabilitation Hospital and performed in accordance with the Declaration of Helsinki. All subjects gave written informed consent in accordance with the Declaration of Helsinki. The protocol was approved by the local institutional review board. Healthy subjects’ cohort data have been published elsewhere.

## Author Contributions

AT, CR, AD, and AH collected data. AT and CR analyzed the data, interpreted the results, and drafted the manuscript. FF designed the study, interpreted the results, and critically reviewed the manuscript. AD, AH, JM-Q, JP, and SF critically reviewed the manuscript and had substantial intellectual contributions. All authors approved the last version of the manuscript.

## Conflict of Interest Statement

The authors declare that the research was conducted in the absence of any commercial or financial relationships that could be construed as a potential conflict of interest.
