# Supplementary material for: Effects of Transcranial Direct Current Stimulation, Transcranial Pulsed Current Stimulation, and Their Combination on Brain Oscillations in Patients with Chronic Visceral Pain: A Pilot Crossover Randomized Controlled Study
Source: Front Neurol. 2017 Nov 1;8:576. doi: 10.3389/fneur.2017.00576 (PMC5672558; doi:10.3389/fneur.2017.00576)
Supplement: Supplementary file 1 [file table_1.docx]

**Supplemetary material**

| ID | VAS Depression | | | | VAS Stress | | | | VAS Sleepiness | | | | VAS Anxiety | | | |
| --- | --- | --- | --- | --- | --- | --- | --- | --- | --- | --- | --- | --- | --- | --- | --- | --- |
|  | tPCS/ tDCS | tPCS | tDCS | Sham | tPCS/ tDCS | tPCS | tDCS | Sham | tPCS/ tDCS | tPCS | tDCS | Sham | tPCS/ tDCS | tPCS | tDCS | Sham |
| 1 | 0 | -1 | -2 | -1 | -1 | -1 | 0 | -1 | 5 | 3 | 3 | 1 | -4 | 0 | -6 | -2 |
| 2 | 0 | 0 | 0 | 0 | 0 | 0 | 0 | 0 | 0 | 0 | 0 | 0 | 0 | -2 | 0 | 0 |
| 3* | _-_ |  | -1 |  |  |  | 1 |  |  |  | 1 |  |  |  | 3 |  |
| 4 | 2 | 0 | 1 | -0.5 | 0 | 0 | -2 | -0.5 | -2 | 0 | 1 | -2 | 1 | 0 | 0 | 0 |
| 5 | 0 | -3 | 0 | 0 | 0 | 4 | 0,5 | 0 | -3 | 3 | 1 | 0 | 0 | -4 | 0.5 | 0 |
| 6 | 0 | 0 | 0 | 0 | 0 | 0 | 0 | -2 | 0 | 0 | 0 | -2 | 0 | -1 | 0 | 1 |

Table 1: Individual data of the difference (pre- minus post) after tES, for the Visual Analogue Scale (VAS) for depression, stress, sleepinedd and anxiety.

| ID | Von Frey – Painful Side | | | | Von Frey – Non Painful Side | | | | |
| --- | --- | --- | --- | --- | --- | --- | --- | --- | --- |
|  | tPCS/ tDCS | tPCS | tDCS | Sham | tPCS/ tDCS | tPCS | tDCS | Sham |  |
| 1 | -0.27 | -0.1 | -0.1 | 0.1 | 0.00 | 0.05 | 0.08 | -0.14 |  |
| 2 | -0.32 | 0.45 | 0.05 | 0.00 | -0.11 | 0.07 | 0.09 | 0.04 |  |
| 3* |  |  | 0.04 |  |  |  | -0.13 |  |  |
| 4 | -0.02 | -0.00 | -0.02 | 0.02 | 0.02 | 0.0 | -0.02 | 0 |  |
| 5 | -0.12 | 0.52 | -2.13 | 0.07 | -0.0 | -0.12 | -28.2 | -0.04 |  |
| 6 | 0 | -0.87 | 0.04 | 0.4 | 5.23 | -0.04 | -0.11 | 0.05 |  |

Table 2: Individual data of the difference (pre- minus post) after tES, for the Von Frey assessment.

| ID | PPT | | | | CPM | | | |
| --- | --- | --- | --- | --- | --- | --- | --- | --- |
|  | tPCS/ tDCS | tPCS | tDCS | Sham | tPCS/ tDCS | tPCS | tDCS | Sham |
| 1 | -4.2 | -2.23 | 0.3 | -4.73 | 0.53 | 3.6 | -1.37 | 0.3 |
| 2 | 2.23 | 2.73 | -1.67 | -2.33 | -1.03 | -1.13 | 1.7 | 0.77 |
| 3* |  |  | -1.23 |  |  |  | 0.1 |  |
| 4 | -1.3 | -2.57 | 0.2 | 1.9 | 0.27 | 3.43 | 0.17 | -3.1 |
| 5 | 0.1 | -2.9 | -1.13 | 0.43 | -3.27 | 1.5 | -0.7 | 0.17 |
| 6 | 1.77 | 1.1 | 1.5 | 4.17 | -3.53 | 0.77 | -0.57 | -4.1 |

Table 3: Individual data of the difference (pre- minus post) after tES, for the Pain Pressure Test (PPT) and Conditioned Pain Modulation (CPM).
