# Supplementary material for: Effects of Transcranial Direct Current Stimulation, Transcranial Pulsed Current Stimulation, and Their Combination on Brain Oscillations in Patients with Chronic Visceral Pain: A Pilot Crossover Randomized Controlled Study
Source: Front Neurol. 2017 Nov 1;8:576. doi: 10.3389/fneur.2017.00576 (PMC5672558; doi:10.3389/fneur.2017.00576)
Supplement: Supplementary file 2 [file table_4.docx]

| *Theta* |  | | | |  | | | |  | | | | |  | | | | |  |  |
| --- | --- | --- | --- | --- | --- | --- | --- | --- | --- | --- | --- | --- | --- | --- | --- | --- | --- | --- | --- | --- |
|  | **tPCS/tDCS** | | | | **tPCS** | | | | **tDCS** | | | | | **Sham** | | | | |  |  |
| ID | Pre | | Post | | Pre | | Post | | Pre | | Post | | | Pre | | Post | | |  |  |
| 1 | 0.018717 | | 0.041133 | | 0.015567 | | 0.023167 | | 0.017367 | | 0.02395 | | | 0.020933 | | 0.01615 | | |  |  |
| 2 | 0.016233 | | 0.016317 | | 0.029717 | | 0.041117 | | 0.025167 | | 0.024383 | | | 0.03775 | | 0.063367 | | |  |  |
| 3* |  | |  | |  | |  | | 0.045367 | | 0.058167 | | |  | |  | | |  |  |
| 4 | 0.260833 | | 0.162833 | | 0.236033 | | 0.189383 | | 0.31195 | | 0.196217 | | | 0.154633 | | 0.185183 | | |  |  |
| 5 | 0.146133 | | 0.091383 | | 0.134383 | | 0.138417 | | 0.624467 | | 0.3608 | | | 0.273083 | | 0.0427 | | |  |  |
| 6 | 0.061083 | | 0.067383 | | 0.063333 | | 0.039317 | | 0.048083 | | 0.035633 | | | 0.03855 | | 0.041033 | | |  |  |
| *Alpha* | |  | | | |  | | | |  | | | | |  | | | | | |
|  | | **tPCS/tDCS** | | | | **tPCS** | | | | **tDCS** | | | | | **Sham** | | | | | |
| ID | | Pre | | Post | | Pre | | Post | | Pre | | | Post | | Pre | | | Post | | |
| 1 | | 0.08055 | | 0.041133 | | 0.054 | | 0.080883 | | 0.0610167 | | | 0.06175 | | 0.041067 | | | 0.020767 | | |
| 2 | | 0.033633 | | 0.0712 | | 0.1044 | | 0.143183 | | 0.0287167 | | | 0.02078 | | 0.11135 | | | 0.159833 | | |
| 3* | |  | |  | |  | |  | | 0.1205833 | | | 0.12288 | |  | | |  | | |
| 4 | | 0.137083 | | 0.0815 | | 0.095667 | | 0.05845 | | 0.1124 | | | 0.05758 | | 0.07395 | | | 0.0752 | | |
| 5 | | 0.2515 | | 0.1753 | | 0.3209 | | 0.232317 | | 0.9122167 | | | 0.40055 | | 0.5998 | | | 0.089867 | | |
| 6 | | 0.138883 | | 0.124217 | | 0.21895 | | 0.109567 | | 0.11355 | | | 0.11898 | | 0.073183 | | | 0.07865 | | |
| *Low Alpha* | |  | | | |  | | | |  | | | | |  | | | | |  |
|  | | **tPCS/tDCS** | | | | **tPCS** | | | | **tDCS** | | | | | **Sham** | | | | |  |
| ID | | Pre | | Post | | Pre | | Post | | Pre | | Post | | | Pre | | Post | | |  |
| 1 | | 0.142533 | | 0.072317 | | 0.083783 | | 0.154333 | | 0.0911 | | 0.105233 | | | 0.057283 | | 0.0328 | | |  |
| 2 | | 0.033383 | | 0.068633 | | 0.1374 | | 0.20315 | | 0.02765 | | 0.021667 | | | 0.164133 | | 0.28185 | | |  |
| 3* | |  | |  | |  | |  | | 0.1213 | | 0.145517 | | |  | |  | | |  |
| 4 | | 0.2243 | | 0.144633 | | 0.164367 | | 0.100783 | | 0.214483 | | 0.099417 | | | 0.125 | | 0.134483 | | |  |
| 5 | | 0.30485 | | 0.27325 | | 0.4161 | | 0.391117 | | 1.111817 | | 0.520367 | | | 0.976883 | | 0.14485 | | |  |
| 6 | | 0.150817 | | 0.111333 | | 0.15605 | | 0.090717 | | 0.098233 | | 0.092717 | | | 0.058633 | | 0.0631 | | |  |
| *High Alpha* | |  | | | |  | | | |  | | | | |  | | | | | |
|  | | **tPCS/tDCS** | | | | **tPCS** | | | | **tDCS** | | | | | **Sham** | | | | | |
| ID | | Pre | | Post | | Pre | | Post | | Pre | | | Post | | Pre | | Post | | | |
| 1 | | 0.040883 | | 0.021167 | | 0.034933 | | 0.033933 | | 0.0418 | | | 0.0339 | | 0.0307 | | 0.013067 | | | |
| 2 | | 0.033817 | | 0.072833 | | 0.0833 | | 0.104817 | | 0.02941667 | | | 0.020233 | | 0.07755 | | 0.08175 | | | |
| 3* | |  | |  | |  | |  | | 0.1201 | | | 0.1084 | |  | |  | | | |
| 4 | | 0.0813 | | 0.041117 | | 0.051717 | | 0.03135 | | 0.04706667 | | | 0.030767 | | 0.04125 | | 0.037283 | | | |
| 5 | | 0.21735 | | 0.11255 | | 0.259967 | | 0.130683 | | 0.78453333 | | | 0.3239 | | 0.35845 | | 0.054683 | | | |
| 6 | | 0.13125 | | 0.132383 | | 0.25925 | | 0.121667 | | 0.12331667 | | | 0.1358 | | 0.082533 | | 0.0886 | | | |

| *High Beta* |  | |  | |  | | |  | | |
| --- | --- | --- | --- | --- | --- | --- | --- | --- | --- | --- |
|  | **tPCS/tDCS** | | **tPCS** | | **tDCS** | | | **Sham** | | |
| ID | Pre | Post | Pre | Post | Pre | Post | | Pre | Post | |
| 1 | 0.003183 | 0.00335 | 0.006333 | 0.004917 | 0.006733 | 0.004933 | | 0.009917 | 0.003167 | |
| 2 | 0.0032 | 0.0037 | 0.004433 | 0.005433 | 0.004033 | 0.004017 | | 0.00505 | 0.00465 | |
| 3* |  |  |  |  | 0.017467 | 0.02025 | |  |  | |
| 4 | 0.0001 | 0 | 0.0001 | 0.0001 | 0.0001 | 0.0001 | | 0.000133 | 0.00000 | |
| 5 | 0.000117 | 0.000117 | 0.000217 | 0.0001 | 0.001067 | 0.001317 | | 0.000183 | 0.00067 | |
| 6 | 0.00015 | 0.000167 | 0.000217 | 0.0001 | 0.000217 | 0.0001 | | 0.000217 | 0.0001 | |
| *Ratio* |  | |  | |  | | |  | | |
|  | **tPCS/tDCS** | | **tPCS** | | **tDCS** | | | **Sham** | | |
| ID | Pre | Post | Pre | Post | Pre | | Post | Pre | | Post |
| 1 | 4.375133 | 1.00615 | 3.403233 | 3.557067 | 3.56115 | | 2.492833 | 2.07865 | | 1.330617 |
| 2 | 2.063133 | 4.38375 | 3.56515 | 3.533583 | 1.1467833 | | 0.877 | 2.980917 | | 2.576433 |
| 3* |  |  |  |  | 2.5095 | | 2.147567 |  | |  |
| 4 | 0.5258 | 0.504033 | 0.404017 | 0.30895 | 0.36605 | | 0.29915 | 0.477 | | 0.407167 |
| 5 | 1.7183 | 1.92295 | 2.387 | 1.748567 | 1.4653 | | 1.102983 | 2.180283 | | 1.983117 |
| 6 | 2.26835 | 1.839583 | 3.474467 | 2.7906 | 2.3691833 | | 3.339517 | 1.906567 | | 1.91235 |
| *Peak* |  | |  | |  | | |  | | |
|  | **tPCS/tDCS** | | **tPCS** | | **tDCS** | | | **Sham** | | |
| ID | Pre | Post | Pre | Post | Pre | Post | | Pre | Post | |
| 1 | 9.286433 | 4.907917 | 9.2253 | 9.08275 | 9.062433 | 9.1642 | | 9.388233 | 8.308883 | |
| 2 | 10.50832 | 10.7527 | 9.7548 | 9.5919 | 10.46757 | 7.127717 | | 9.734467 | 9.4697 | |
| 3* |  |  |  |  | 10.101 | 9.876983 | |  |  | |
| 4 | 7.6979 | 7.8201 | 7.433167 | 7.229567 | 7.5758 | 7.127733 | | 7.453567 | 7.2092 | |
| 5 | 9.51045 | 8.655067 | 10.38613 | 8.634767 | 9.245667 | 8.5533 | | 8.756967 | 8.777283 | |
| 6 | 9.204983 | 9.89735 | 11.26183 | 10.83415 | 11.24142 | 10.8749 | | 11.77093 | 10.7527 | |

Table 4: Individual data of pre- and post-tES power spectrum in the different bandwidths in the central region.
