# Supplementary material for: Effects of Transcranial Direct Current Stimulation, Transcranial Pulsed Current Stimulation, and Their Combination on Brain Oscillations in Patients with Chronic Visceral Pain: A Pilot Crossover Randomized Controlled Study
Source: Front Neurol. 2017 Nov 1;8:576. doi: 10.3389/fneur.2017.00576 (PMC5672558; doi:10.3389/fneur.2017.00576)
Supplement: Supplementary file 3 [file table_5.docx]

| ID | Condition | **Theta** | | **Alpha** | | **Low alpha** | | **High alpha** | |
| --- | --- | --- | --- | --- | --- | --- | --- | --- | --- |
|  |  | **pre** | **post** | **pre** | **post** | **pre** | **post** | **pre** | **post** |
| 1 | tPCS/tDCS | 0.0818 | 0.0886 | 0.1818 | 0.0259 | 0.1563 | 0.0130 | 0.19745 | 0.03375 |
|  | tPCS | 0.04725 | 0.07965 | 0.1893 | 0.21305 | 0.26765 | 0.35155 | 0.1411 | 0.1278 |
|  | tDCS | 0.04325 | 0.06565 | 0.1565 | 0.1587 | 0.18665 | 0.1716 | 0.1379 | 0.15075 |
|  | Sham | 0.02195 | 0.09395 | 0.0211 | 0.06335 | 0.01635 | 0.04905 | 0.0241 | 0.07215 |
| 2 | tPCS/tDCS | 0.1859 | 0.23445 | 0.17535 | 0.27165 | 0.12495 | 0.31595 | 0.14415 | 0.2444 |
|  | tPCS | 0.27655 | 0.2135 | 0.31055 | 0.23855 | 0.40545 | 0.3721 | 0.25215 | 0.15645 |
|  | tDCS | 0.2076 | 0.2102 | 0.14105 | 0.1276 | 0.16715 | 0.1888 | 0.12495 | 0.0900 |
|  | Sham | 0.2283 | 0.1515 | 0.2473 | 0.2331 | 0.2783 | 0.28525 | 0.2282 | 0.2010 |
| 3* | tDCS | 0.11095 | 0.0848 | 0.0905 | 0.04475 | 0.28295 | 0.17395 | 0.05245 | 0.0330 |
| 4 | tPCS/tDCS | 0.33435 | 0.59095 | 0.28805 | 0.5062 | 0.3072 | 0.53835 | 0.27625 | 0.4864 |
|  | tPCS | 0.4449 | 0.5907 | 0.2653 | 0.47135 | 0.3853 | 0.5251 | 0.19145 | 0.4382 |
|  | tDCS | 0.61785 | 0.45285 | 0.4114 | 0.3524 | 0.47905 | 0.33885 | 0.3697 | 0.36065 |
|  | Sham | 0.50705 | 0.4297 | 0.31545 | 0.2451 | 0.38595 | 0.29735 | 0.2721 | 0.21295 |
| 5 | tPCS/tDCS | 0.80015 | 0.71955 | 0.7125 | 0.3856 | 0.64405 | 0.4192 | 0.65465 | 0.49835 |
|  | tPCS | 0.18205 | 0.1046 | 0.2888 | 0.29505 | 0.139 | 0.11885 | 0.1347 | 0.07395 |
|  | tDCS | 0.2667 | 0.07495 | 0.2758 | 0.1773 | 0.16065 | 0.11965 | 0.19125 | 0.13605 |
|  | Sham | 0.18945 | 0.1010 | 0.4958 | 0.15205 | 0.3213 | 0.1637 | 0.16385 | 0.0281 |
| 6 | tPCS/tDCS | 0.32015 | 0.51175 | 0.4605 | 0.6067 | 0.37045 | 0.46225 | 0.51585 | 0.69555 |
|  | tPCS | 0.3395 | 0.27515 | 0.57635 | 0.40835 | 0.3818 | 0.2166 | 0.6961 | 0.5263 |
|  | tDCS | 0.34135 | 0.5488 | 0.50615 | 0.69135 | 0.3507 | 0.57295 | 0.6018 | 0.76425 |
|  | Sham | 0.27615 | 0.38745 | 0.38635 | 0.46145 | 0.2465 | 0.2728 | 0.47245 | 0.5775 |

Table 5: Individual data of pre- and post-tES interhemispheric frontal coherence.
